# Overexpression of High-Mobility Motor Box 1 in the Blood and Tissues of Patients with Head and Neck Squamous Cell Carcinoma

**Published:** 2018-09

**Authors:** Farnaz Mohajertehran, Hossein Ayatollahi, Kamran Khazaeni, Mohammad-Taghi Shakeri, Nooshin Mohtasham

**Affiliations:** 1 *Dental Research Center, Mashhad University of Medical Sciences, Mashhad, Iran* *.*; 2 *Cancer Molecular Pathology Research Center, Faculty of Medicine, Mashhad University of Medical Sciences, Mashhad, Iran.*; 3 *Department of Otorhinolaryngology, Mashhad University of Medical Sciences, Mashhad, Iran.*; 4 *Department of Biostatic, Mashhad University of Medical Sciences, Mashhad, Iran. *

**Keywords:** Biomarker, Early diagnosis, Head and neck squamous cell carcinoma (HNSCC), High-mobility motor box (HMGB1), Lymph node involvement

## Abstract

**Introduction:**

Head and neck squamous cell carcinoma (HNSCC) is one of the most common cancers in the world. Extra- and intra-cellular high-mobility motor box 1 (HMGB1) proteins are invovled in the pathogenesis and prognosis of cancer. Regarding this, the present study was conducted with the aim of investigating the expression of HMGB1 protein and mRNA levels in the blood, tumor tissue, and marginal normal tissue of patients with head and neck squamous cell carcinoma (HNSCC) using the quantitative real-time polymerase chain reaction (qRT-PCR) and immunohistochemistry (IHC).

**Materials and Methods:**

This study was performed on 88 patients with HNSCC, who referred to the otorhinolaryngology and oral pathology departments, affiliated to Mashhad University of Medical Sciences, Mashhad, Iran, and a group of healthy subjects (i.e., control group) matched in terms of age and gender. RNA was collected from fresh tumor tissues, marginal tissues, and blood, followed by the implementation of quantitative PCR on the specimens. Furthermore, the expression of HMGB1 in tumor and normal margins was evaluated by means of IHC. The data were analyzed in SPSS software.

**Results:**

According to the results the expression levels of HMGB1 protein and mRNA were significantly higher in the tumor tissue than in the normal margin tissues (P<0.01). In addition, there was a significant correlation between histologic grading and the expression of HMGB1 protein and mRNA in tissues (P<0.05). Furthermore, the receiver operating characteristic curve of the HMGB1 mRNA in tissue was located closer to the theoretical 100% sensitivity.

**Conclusion:**

The findings revealed a higher increase in the levels of mRNA and HMGB1 protein in HNSCC, compared to those in the normal margin tissues. In addition, HMGB1 mRNA showed a significant expression in the tissue and blood of the patients with lymph node involvement.

## Introduction

Squamous cell carcinoma (SCC) is the most common head and neck malignancy. Accordingly, head and neck squamous cell carcinoma (HNSCC) is one of the most recurrent cancers that is among the top 10 causes of mortality in the world. The late diagnosis of HNSCC can be due to several reasons, including its lack of symptom in early stages, similar clinical features to other lesions, and variety of clinical forms. On the other hand, SCC is the 6^th^ and 12^th^ cancer in males and females, respectively. Annually, more than 400,000 new cases are diagnosed with SCC, resulting in 200,000 deaths. Despite the development of invasive therapeutic approaches for HNSCC, this disease still has a relatively weak prognosis (1,2).

Given the unsatisfactory treatment outcomes of HNSCC patients, researchers are greatly interested in the identification of the biomarkers that can be used for the early diagnosis or prognosis of this disease. In general, HNSCC can be categorized based on anatomical tumor location. The main cause of this cancer is the mucosal destruction of the oral cavity, sinus nasal tract, nasopharynx, larynx, pharynx, oropharynx, and hypopharynx. 

The major treatment approaches for HNSCC include surgery, chemotherapy, and radiotherapy. Delayed diagnosis and insufficient response to the treatment of the tumor lead to the enhancement of HNSCC-related mortality. Despite the availability of advanced therapeutic methods, HNSCC results in a high morbidity rate with a 5-year survival rate of about 50%. Given the insufficient findings on this disease, researchers have paid special attention to the recognition of the biomarkers that contribute to the early detection of the condition (3-5). 

According to the literature, high-mobility group box 1 (HMGB1) protein is a highly protected nucleic and extracellular protein in the eukaryotic cells. Transcription regulation necessitates the presence of HMGB1 in many cancer genes (6-9). The HMGB1 serves two main functions in the extracellular matrix and nucleus through acting as a DNA-binding protein and transcription factor, regulating gene expression. In addition, HMGB1 is a highly protected nuclear protein that functions as a chromatin-binding agent to bind DNA and increases access to transcription proteins for specific DNA targets (10-12). In addition to its nuclear role, HMGB1 acts as an extracellular signal molecule during inflammation, cell differentiation, cell migration, and tumor metastasis. Extracellular HMGB1 is both recognized as a “danger signal” and “inflammatory mediator”. Moreover, it is released from living inflammatory, stressed, or necrotic cells by active secretion and passive release (13-16). This protein plays an important role in biological processes, such as transcription, DNA repair, and extracellular signaling (17). Generally, HMGB1 contains the A- and B-box (two DNA-binding domains) with a negative C-terminal tail and 215 amino acid polypeptides (18,19). 

The HMGB1 is inactively released from the necrotic cells and actively secreted by the inflammatory cells. Furthermore, this protein is absorbed by several receptors, including receptors for advanced relation products, toll-like receptors (TLR)-2, TLR-4, TLR-9, and CD24, eliciting negative regulatory signals. In general, inflammation is increased through the response of HMGB1 to infection and damage (20-24). Under normal conditions, most of HMGB1s are focused on the core group. Nuclear HMGB1 binds to DNA to control gene transcription and facilitate DNA repair as a response to chromatin remodeling. 

For example, HMGB1 is a transcriptional cofactor of p53, p73, and retinoblastoma protein. In addition, HMGB1 is identified as a damage-associated molecular pattern during the cell death, inflammation, and exposure to environmental stressors ( 16 , 20 , 21 , 25 ). The HMGB1 has two important roles in cancer development, progression, and therapy (14, 26). Meanwhile, different roles have been attributed to extracellular and intracellular HMGB1s in cancer. Extracellular HMGB1 acts as a pro-tumor protein due to its cytokine, chemokine, and growth factor action, whereas intracellular HMGB1 increases drug resistance due to its proautophagic activity (27,28). 

However, there is no accurate data regarding the functional role of intracellular and extracellular HMGB1s in tumorigenesis (14, 26-28). With this background in mind, the present study aimed to evaluate the expression of HMGB1 protein and mRNA in the tumor tissues and normal margin tissues of HNSCC patients and the blood samples of healthy individuals with matched age and gender. In addition, the expressions of cytoplasmic and nuclear HMGB1 were compared in patients with different disease stages and grades. Moreover, HMGB1 mRNA and protein molecule expressions were assessed in the tissue and blood samples to identify their potentiality to be used as a marker facilitating the early diagnosis of the disease.

## Materials and Methods

*Study Population: *This study was conducted on the tumor and normal margin tissues of 44 patients with HNSCC (i.e., 33 males and 11 females) with the mean age of 59.6±13.2 years (age range: 27-83 years) referring to the otorhinolaryngology and oral pathology departments, affiliated to Mashhad University of Medical Sciences, Mashhad, Iran, during September 2015-June 2017. The SCC was confirmed in all patients based on histologic examinations. The staging was determined based on the tumor-node-metastasis staging system, where grades I and II were interpreted as early stages, and grades III and VI were recognized as advanced stages (29). 

This clinical study was approved by the Ethics Committee of Mashhad University of Medical Sciences, Mashhad, Iran. Written informed consents were obtained from all subjects prior to the research. The exclusion criterion included undergoing surgery, chemotherapy, and radiotherapy. It is noteworthy that the histologic diagnosis of the patients was confirmed by a pathologist. Table 1 tabulates the clinical and pathological properties of all patients. As the control group, blood samples were collected from the clients referring to Qaem Hospital in Mashhad for annual checkup. They were normal in terms of the response to hematological tests and matched with the case group (i.e., patients under study) in terms of the age and gender

**Table 1:** Clinicopathological characteristics of patients with head and neck squamous cell

Characteristic		N	%
**Patients**		44	100
**Age**	Mean age: 59.61±13.22		
	≤59	23	52.27
	>59	21	47.73
**Gender**			
	Male	33	75.00
	Female	11	25.00
**Tumor stage**			
	Early	19	43.18
	Advanced	25	56.82
**Lymph node involvement**			
	Absent	23	54.54
	Present	21	47.72
**Histopathologic grade**			
	I	27	61.36
	II	14	31.82
	III	3	6.82
**Tumor position**			
	Lip	10	22.72
	Tongue	14	31.82
	Larynx	16	36.36
	Nasal cavity	2	4.55
	Ear	2	4.55

*Quantitative Polymerase Chain Reaction and RNA Isolation from Tissue Samples*

 First, RNA was extracted from the tumor and normal margin tissues, which led to the formation of complementary DNA (cDNA). Subsequently, 20-30 mg fresh tumor and normal tissues were collected and submerged in RNAlater Stabilization Reagent (Qiagen, Germany) at -80ºC. In addition, the extraction of total RNA from the fresh tissues was accomplished using the High Pure RNA Tissue Kit (Roch, Germany) following the manufacturer’s instructions. In the next stage, 200 ng of RNA was reverse-transcribed with Oligo dT at 65ºC for 5 min in 20 μl reaction volume by means of the RevertAid First Strand cDNA Synthesis Kit(Thermos Scientific, Germany). 

Subsequently, the cDNA was subjected to real-time PCR (RT-PCR) using the SYBR Green Real-Time PCR Master Mix Kit (Thermos Scientific, Germany) and 7500 Fast Real-Time PCR System (Applied Biosystems, USA). The primer sequences used for PCR included β-actin F-5'-AGCGGGAAATCGT GCGTG-3', R-5'-GGGTACATGGTGGTGCC G-3', HMGB1 F-5'-CTCAGAGAGGTG GAAGACCATGT-3',and RGGGATATAGGT TTTCATTTCTCTTTC-3'.

Furthermore, the PCR conditions entailed 10 min at 95ºC (hot-start enzyme activation), 45 cycles of denaturation for 15 sec at 95ºC, annealing for 1 min at 60ºC, and extension for 30 sec at 72ºC. Three normal tissues were collected from three donors as healthy controls. It should be noted that two duplications of PCR experiments were performed in this research. In this regard, the delta-delta CT (ΔΔCT) method was used for gene expression, and also for RT-PCR data analysis by considering the HMGB1 as the target gene and beta-actin gene as the control gene.

*Quantitative Polymerase Chain Reaction and RNA Isolation from Blood Samples*

In this research, bloodletting was carried out on all HNSCC patients and their age- and gender-matched counterparts in the control group using EDTA tubes. Moreover, Ficoll gradient centrifugation method was used to separate the peripheral blood mononuclear cell by Sigma Aldrich kit (Sigma Aldrich Company, USA). Afterwards, the samples were subjected to RNA extraction using the TriPure solution (TriPure kit, Germany). All test processes were carried out according to the kit instructions. 

On the other hand, RevertAid™ H Minus First Strand cDNA Synthesis Kit (Thermo Fisher Scientific, USA) was utilized for cDNA synthesis. Beta-actin gene control primers were assessed for cDNA confirmation by PCR method, and the results were checked on agarose. The synthesis of cDNA was performed at a wavelength of 260 nm using NanoDrop instrument (Thermo Scientific 2000, Finland). The Fast Real-Time PCR System (ABI, USA) was also used in this experiment. 

In the present study, the samples were tested in two replications. The RT-PCR Master Mix (20 µL for each reaction) contained 10 µL SYBR Green master mix, 0.5 µL ROX stain, 1 µL of each forward and reverse primers (10 pm), 2 µL cDNA, and 5.5 µL double distilled sterile water. The planned temperature patterns in the thermocycler included 95Cº for 10 min, 95Cº for 15 sec, 60ºC for 1 min, and 72ºC for 30 sec. The second, third, and fourth steps were repeated 45 times, and the quantity of gene expression was calculated using the Δ∆CT method. Furthermore, melting curve analysis was performed to confirm the specificity of the products‏. 

*Histological and Immunohistochemical Procedures*

All tumor and normal margin tissues were fixed in formalin and embedded in paraffin. In the next stage, all samples were cut (2-4 μm) and stained with haematoxylin and eosin using the standard histochemical procedures. Subsequently, the sections were dewaxed in xylene, and tissue sections were rehydrated by changing the ethanol concentration. The samples were then blocked with 0.3% hydrogen peroxide, and antigen retrieval was performed through treatment by microwave with 10 mM citrate buffer (pH 6.0) for 30 min, followed by cooling the samples at room temperature. Afterwards, the sections were washed with phosphate buffered saline and incubated at 4ºC overnight with the monoclonal HMGB1 antibody (J2E1-sc-135809) using a 1:100 dilution (Santa Cruze Biotechnology, USA). In the next phase, the secondary antibody-conjugated horseradish peroxidase was applied, followed by the development and counterstaining of diaminobenzidine tetrahydrochloride and hematoxylin. Moreover, the colorectal cancer samples obtained as positive for HMGB1 were considered as positive controls. 

*Evaluation of Staining Procedure*

In this study, the immunohistochemic ally-stained tissue samples were scored by two pathologists by means of a light microscope equipped with objectives with 400X magnification. Five filed in each slide were selected for the estimation of the HMGB1 expression, which was scored based on the number of positive cells and intensity. The positive cells were categorized into five groups of zero (0%), one (≤25%), two (26-50%), three (51-74%), and four (≥75%) based on the percentage. In addition, the intensity of nuclear or cytoplasmic staining was classified into four groups of no staining/background of negative controls (score=1), weak staining (score=2), moderate staining (score=3), and intense staining (score=4). The final indicator was obtained by summing up the intensity and percentage of cellularity, followed by the categorization of the scores. In this regard, a score range of 0-4 was considered as low expression, whereas a score range of 5-7 was regarded as high expression (30).

*Statistical analysis*

Student’s t-test and Pearson’s Chi-square test were exploited to evaluate the correlation between the HMGB1 mRNA levels detected in the peripheral blood and tumor tissue samples. In this regard, the clinicopathological characteristics were considered as well. Additionally, Wilcoxon signed-rank and Chi-square tests were applied to examine the correlation between the expression of HMGB1 protein in the tumor and healthy normal tissues.

The receiver operating curve (ROC) analysis was performed, and the area under the curve was calculated separately for each of the two samples of tissue and blood for this marker. Furthermore, the ROC was used to show sensitivity and specificity. Data analysis was carried out in SPSS software (version 11.5, Chicago, IL, USA). P-value less than 0.05 was considered statistically significant.

## Results

*High-Mobility Motor Box 1 Protein Levels in Tumor and Normal Tissues by Immunohisto- chemistry*

**Fig 1:**
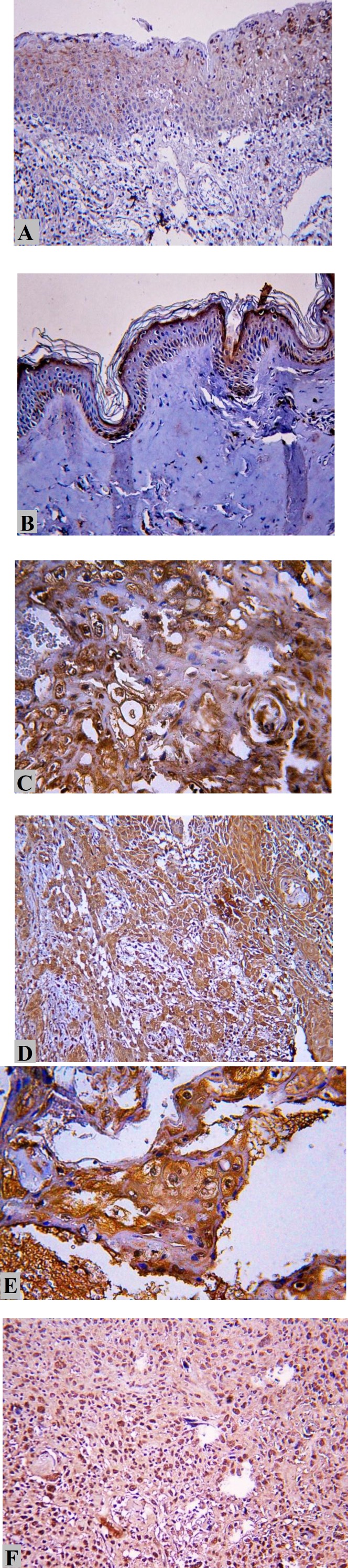
High-mobility motor box 1 expression in human endometrial tissues by immunohistochemistry, A) 200X magnification: no expression in the normal tissue, B) 200X magnification: no expression in the basal layer of the normal tissue, C) 400X magnification: high cellular and nuclear expression (SCC grade III), D) 400X magnification: high cellular expression (SCC grade III), E) 200X magnification: high nuclear expression of HMGB1 (SCC grade III), and F) 200X magnification: Nuclear expression of HMGB1 in control sample (Squamous Cell Carcinoma), 200X magnification

After the staining of all tumor and normal margin tissues, the results obtained through pathology and immunohisto- chemistry (IHC) were confirmed by the two pathologists. Table 2 presents the levels of HMGB1 expression in the tumor and normal tissue samples. The positive expression of HMGB1 in HNSCC tissues was demonstrated through brown staining in the cytoplasm or nuclei or both cytoplasm and nuclei. Figure 1 displays the results of HMGB1 expression in the tumor and normal tissues. 

**Table 2:** Expression of high-mobility motor box 1 in tumor and healthy normal tissues

		Tumor tissue		Healthy tissue		P-value
		N	%	N	%	
**HMGB1 staining** **cellularity **	0%	0	0	28	63.6	<0.001
	≤25%	0	0	4	9.1	
	26-50%	17	38.6	10	22.7	
	51-74%	16	36.4	1	2.3	
	≥75%	11	25.0	1	2.3	
**HMGB1 staining** **intensity **	Score 1	0	0	28	63.6	<0.001
	Score 2	30	68.2	14	31.8	
	Score 3	13	29.5	2	4.5	
	Score 4	1	2.3	0	0	
**HMGB1 Protein location expression in tissue**	No expression	0	0	28	63.6	<0.05
	Cytoplasmic expression (C)	32	72.7	16	36.4	
	Nucleus expression (N)	2	4.5	0	0	
	C & N	10	22.7	0	0	
**Expression**	Low expression	13	29.5	41	93.2	<0.001
	High expression	31	70.5	3	6.8	

HMGB1: high-mobility motor box 1

*Evaluation of High-Mobility Motor Box 1 mRNA Levels in Tissue and Blood Samples by Quantitative Real-Time Polymerase Chain Reaction*

**Fig 2:**
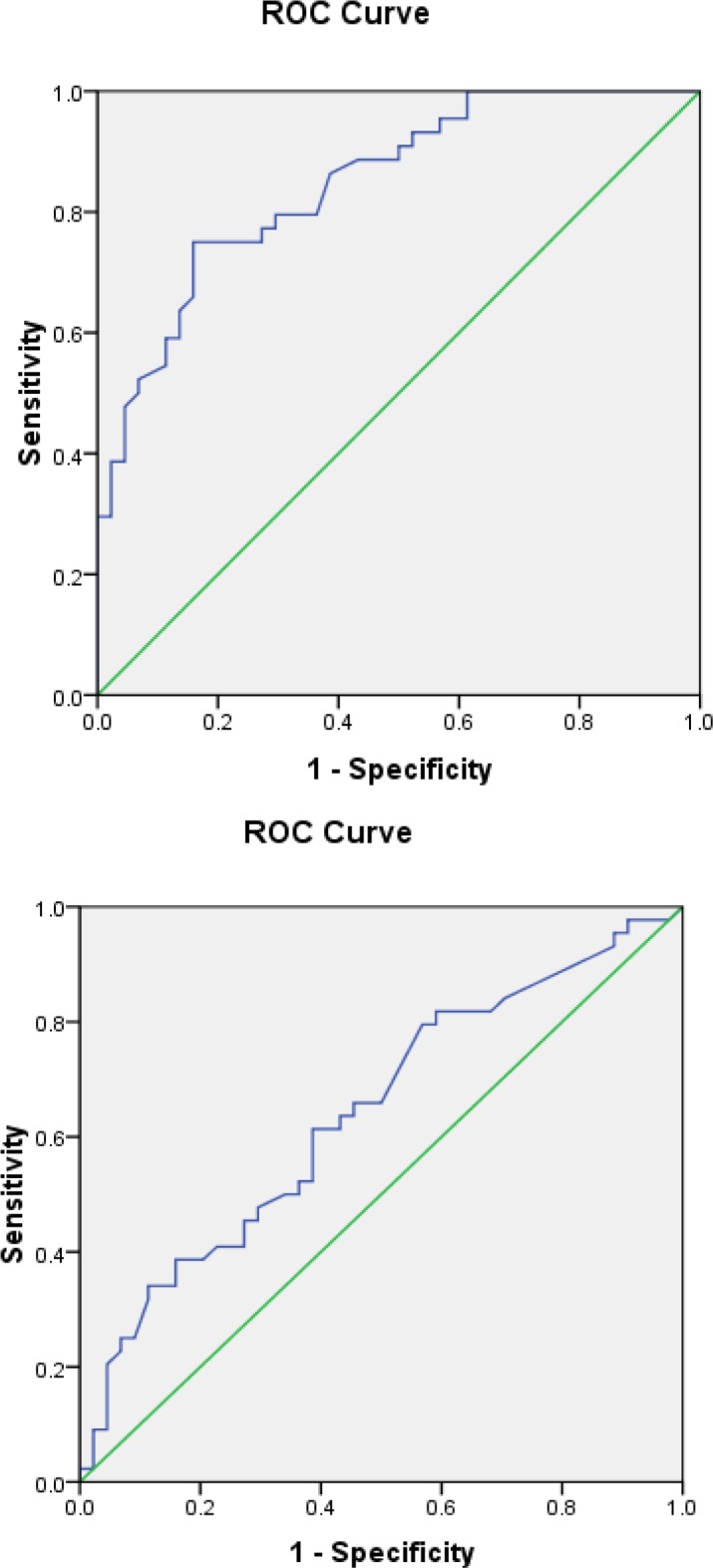
Receiver operating characteristic curves of high-mobility motor box 1 mRNA expression in the tissue and blood samples

The peripheral blood and tumor tissue RNA samples of 44 patients were analyzed by quantitative RT-PCR for *HMGB1* gene. The peripheral blood samples were obtained from 44 healthy individuals, who were matched with patients in terms of age and gender. Table 3 demonstrates the *HMGB1* transcript levels in the peripheral blood and tumor tissue samples for all HNSCC patients and the control group. Figure 2 illustrates the distribution of *HMGB1* mRNA levels in the tissue and blood samples obtained from the patients and controls. 

**Table 3:** Distribution of high-mobility motor box 1 mRNA levels in the patients and controls

**mRNA gene expression**	N	%
Healthy control		
HMGB1 tissue (T) (+)	15	34.1%
HMGB1 blood (B) (+)	17	38.6
Patients		
HMGB1 T(+)	40	90.1
HMGB1 T(+) & B(-)	19	43.2
HMGB1 B(+)	22	50.0
HMGB1 B(+) & T(-)	0	0
HMGB1 B & T(+)	21	47.7
HMGB1 B & T(-)	1	0.22

HMGB1: High-mobility motor box 1

**Table 4:** Diagnostic performance of high-mobility motor box 1 in the tissue and blood samples by quantitative real-time polymerase chain reaction

	HMGB1 Tissue	HMGB1Blood
**Sensitivity %**	90.9%	38.6%
**Specificity %**	65.9%	79.5%
**PPV %**	61.5%	65.4%
**NPV %**	87.9%	56.5%

HMGB1: high-mobility motor box 1, PPV: positive predictive value, NPV: negative predictive value

Table 4 shows the measurement of the diagnostic performance of HMGB1 in the tissue and blood samples in terms of sensitivity and specificity. In the tissue samples, the sensitivity and specificity of HMGB1 were obtained as 90.9% and 65.9%, respectively. On the other hand, regarding the blood samples, these values were estimated as 38.6% and 79.5%, respectively. 

*Association of High-Mobility Motor Box 1* *Protein Levels with mRNA Levels in Head and Neck Squamous Cell Carcinoma Tissue*

In this study, the *HMGB1* expression in the HNSCC tumor and normal tissues was evaluated using IHC staining. The expression of HMGB1 protein in the tissue samples was classified in two levels of low and high expressions (Table 2). According to the results, HMGB1 expression was significantly higher in the tumor tissues, compared to that in the normal tissues (P<0.01). In addition, the tumor tissues had a significantly higher *HMGB1* mRNA expression level as compared to the healthy tissues in HNSCC patients (P<0.001). Furthermore, there was a significant relationship between the expression levels of HMGB1 protein and mRNA in the tumor and healthy tissues of HNSCC patients (P<0.001). 

*Correlation between High-Mobility Motor Box 1* *Levels and Clinicopathological Characteristics in Patients with Head and Neck Squamous Cell Carcinoma *

Table 5 presents the correlation between HMGB1 expression and the clinicopathological characteristics of the patients with HNSCC. The results revealed a significant correlation between HMGBI protein and mRNA expression in the tissue and histological grading (P<0.05). Furthermore, there was a significant relationship between the disease stage and the expression level of HMGB1 mRNA in the blood (P<0.05). It should be noted that the Mann-Whitney U test was performed to evaluate the significant relationship between the lymph node involvement and HMGB1 mRNA expression level in the tissue and blood patients (P<0.05).

**Table 5:** Correlation between grade, stage, lymph node involvement, the position of the tumor, age, gender and HMGB1 mRNA and protein expression in tissue and blood samples of patients with HNSCC

	Grade	Stage	Lymph node involvement	Position of tumor	Age	Gender
**HMGB1 protein expression in tissue**	0.035	0.111	0.892	0.385	0.532	0.567
**mRNA HMGB1 expression in tissue **	0.008	0.773	0.009	0.951	0.328	0.699
**mRNA HMGB1 expression in blood**	0.055	0.037	0.041	0.432	0.276	0.371

HMGB1: high-mobility motor box 1

*Receiver operating characteristic curve*

The level of HMGB1 mRNA expression in the tissue and blood samples was evaluated by ROC curve. As the results indicated, the ROC curves of HMGB1 mRNA in the tissue were located closer to the theoretical 100% sensitivity. Figure 2 illustrates the specificity and sensitivity of HMGB1 mRNA in the blood and tissue samples. 

## Discussion

*The HMGB1 is a cytokine and a growth* factor, which can be released from tumor cell upon necrosis. In addition, *HMGB1* can lead to chronic inflammation in the microscopic environment of the tumor, as well as tumor cell survival, growth, and metastasis (31). The current study was the first attempt evaluating HMGB1 protein and mRNA levels in the tissue and blood samples of HNSCC patients and a healthy control group. 

In the present study, the potentiality of HMGB1 to be used as a molecular marker for the detection of HNSCC was evaluated and compared. Furthermore, the assessment of the levels of HMGB1 mRNA and HMGB1 protein expression in the peripheral blood and tissue samples of HNSCC patients and normal subjects was accomplished using the quantitative RT-PCR and IHC methods.

The comparison of the expression levels of HMGB1 protein and mRNA in the tissue samples demonstrated that the expression of this gene in the tumor tissue was significantly higher than in the margin of the healthy tissue. On the other hand, the evaluation and comparison of HMGB1 mRNA expression level in the blood of patients and normal subjects demonstrated no significant increase in the expression of this gene in the two groups. Moreover, the results revealed a significant relationship between HMGB1 protein and mRNA expressions in the tissue and histological grading. Our study was the first attempt that simultaneously compared the expression of the mRNA and protein of HMGB1 in the blood and tumor tissue of HNSCC patients and healthy controls. 

Genetic changes resulted in distinct variations in the expression of many genes at both levels of mRNA and protein. In the present study, the samples were subjected to the quantitative RT-PCR due to its high sensitivity in the detection and evaluation of mRNA expression in tumors and cells. 

The main objective of the present study was to provide a quick and non-invasive method with high specificity and sensitivity for the early diagnosis of HNSCC in patients. In the present study, HMGB1 in the tissue was shown to have a good sensitivity (90.9%) and low specificity (50.0%) as a molecular marker. Recent studies have reported a significant increase in the expression level of HMGB1 protein in multiple tumor tissues (32,33). 

In the current study, the expression levels of HMGB1 protein and mRNA were concomitantly assessed in the tissue and blood samples of HNSCC patients and healthy subjects. In addition, to evaluate the potentiality of HMGB1 as an early diagnostic marker for HNSCC, the correlation of HMGB1 expression in the tumor samples with clinical and pathologic factors in patients with HNSCC and healthy controls was estimated, and the sensitivity and specificity of HMGB1 marker were evaluated in the tissue and blood samples. 

In a study performed by Zhang, the expression of HMGB1 protein in four gastric cancer cell lines and non-malignant GES-1 cells was assessed by western blot analysis and immunofluorescence. The results of the mentioned study revealed the overexpression of HMGB1 protein in the cytoplasm of the gastric cancer cells (34), which is consistent with our findings in terms of increased expression. The results of another study conducted by Cheng suggested the level of HMGB1 in serum as a useful marker for the evaluation of tumor stage in hepatocellular carcinoma (18). 

In the current study, the evaluated levels of HMGB1 in the blood and tissue samples of HNSCC patients might be useful for the prediction of lymph node involvement. In another study, a relationship was reported between serum HMGB1 levels and the clinical and pathological parameters of gas chromatography. In the mentioned study, serum HMGB1 levels were demonstrated to be significantly associated with the depth of invasion, lymph node metastasis, tumor size, and poor prognosis (35). 

In another study, the HMGB1 protein was expressed in more than 71.2% of the cancerous tissues and was only non-cancerous in 41.7% of the cancerous tissues. In the mentioned study, the expression of HMGB1 was significantly associated with the clinicopathological characteristics (36), which is in congruence with our findings due to the difference in expression between the tumor and normal tissues. Previous studies have demonstrated the elevation of serum HMGB1 levels in patients with gastric or cervical cancers and hepatocellular carcinoma (18, 35, 36). 

In the present study, mRNA and protein levels of HMGB1 were evaluated in the blood and tissue samples of HNSCC patients. In another study, by Qian investigated HMGB1 protein expression and clinicopathologic characteristics, as well as their association with overall survival time in oropharyngeal squamous cell carcinoma patients. They demonstrated that protein HMGB1 expression in tumor tissue was only significant in tumor staging (37). In the current study, a positive correlation was observed between HMGB1 protein and mRNA levels in tumor samples. Based on our results, the tissue HMGB1 mRNA level can be suggested as a novel diagnostic marker with a high sensitivity for HNSCC tumor tissue. In addition, it is recommended that the levels of HMGB1 in blood be applied to predict lymph node involvement in HNSCC patients. 

## Conclusion

As the findings of the present study indicated, the level of HMGB1 protein was positively correlated with mRNA levels in the tumor samples. Moreover, a positive association was found between the expression of this gene in the blood samples and clinical staging. Our findings suggested the tissue HMGB1 protein and mRNA levels as a novel diagnostic marker with a high sensitivity to define the biologic margin of the tumor. In addition, they can act as an effective marker in pathogenesis and lesion formation. The results were also indicative of the decreased differentiation level of tumor cells by increasing the HMGB1. Consequently, this marker is suggested to be used for the prediction of lymph node involvement. 
